# Metagenomic-based characterization of the gut virome in patients with polycystic ovary syndrome

**DOI:** 10.3389/fmicb.2022.951782

**Published:** 2022-08-16

**Authors:** Liansha Huang, Xiaoling Wu, Shumin Guo, Ying Lv, Peng Zhou, Guangrong Huang, Zuzhen Duan, Wen Sun

**Affiliations:** ^1^Department of Reproductive Health, Shenzhen Bao'an Chinese Medicine Hospital, Guangzhou University of Chinese Medicine, Shenzhen, China; ^2^Department of Acupuncture, Shenzhen Bao'an Chinese Medicine Hospital, Guangzhou University of Chinese Medicine, Shenzhen, China; ^3^Department of Gynecology, Shenzhen Bao'an Chinese Medicine Hospital, Guangzhou University of Chinese Medicine, Shenzhen, China; ^4^Key Laboratory of Health Cultivation of the Ministry of Education, Beijing University of Chinese Medicine, Beijing, China; ^5^Beijing key Laboratory of Health Cultivation, Beijing University of Chinese Medicine, Beijing, China; ^6^School of Traditional Chinese Medicine, Beijing University of Chinese Medicine, Beijing, China

**Keywords:** polycystic ovary syndrome, gut virome, viral dysbiosis, gut bacteriome, whole-metagenome sequencing

## Abstract

**Background:**

Polycystic ovary syndrome (PCOS) is a complex disease that afflicts women of reproductive age, and its pathological mechanism has not been well explained. The gut microbiota is believed to be closely related to the development of PCOS. Although an important component of the gut microbiome, the role of the gut virome in the development of PCOS is still unclear.

**Methods:**

In this study, we profiled and compared the gut viral community of 50 patients with PCOS and 43 healthy women based on the analysis of their fecal whole-metagenome dataset.

**Results:**

The gut virome of PCOS patients exhibited a significant decrease in within-sample viral diversity and a remarkable alteration of the overall virome composition compared with that of healthy controls. At the family level, *Siphoviridae* was significantly depleted in the gut virome of patients, while *Quimbyviridae* was enriched. We identified 1,089 viral operational taxonomic units (vOTUs) that differed in relative abundance between the two groups, of which 455 vOTUs were enriched in PCOS patients (including numerous *Bacteroidaceae* phages) and 634 were enriched in controls (including numerous viruses predicted to infect *Oscillospiraceae*, *Prevotellaceae*, and *Ruminococcaceae*). Functional comparison of the PCOS-enriched and control-enriched vOTUs uncovered the viral functional signatures associated with PCOS. Furthermore, we demonstrated gut viral signatures for disease discrimination and achieved an area under the receiver operator characteristic curve (AUC) of 0.938, demonstrating the potential of the gut virome in the prediction of PCOS.

**Conclusion:**

Our findings reveal specific alterations in viral diversity and taxonomic and functional compositions of the gut virome of PCOS patients. Further studies on the etiology of PCOS and the gut viral community will offer new prospects for treating and preventing PCOS and its related diseases.

## Introduction

Polycystic ovary syndrome (PCOS) is a syndromic female endocrine disease characterized by a disorder of androgen secretion (Patel, 2018), which affects 6–20% of women of reproductive age worldwide (Meier, 2018). The syndrome leads to an increased risk of infertility, high blood pressure, obesity, metabolic disorders, and insulin resistance (Gilbert et al., 2018). Presently, many studies have identified a variety of causes of PCOS, including genetic and environmental factors (Goodarzi et al., 2011; Cui et al., 2013; McAllister et al., 2015; Merkin et al., 2016; Kshetrimayum et al., 2019). However, due to the complexity and heterogeneity of this disease, its exact pathogenesis is still not well known. The gut microbiota has been shown to be involved in host energy metabolism, insulin secretion, and inflammatory responses (Visconti et al., 2019; Lee et al., 2020; Ruff et al., 2020), suggesting that it may play an important role in the development and progression of a variety of diseases (Carding et al., 2015; Fan and Pedersen, 2021). Recent studies have highlighted the potential role of the gut microbiota in influencing the onset and development of PCOS (Qi et al., 2019). Mechanistically, the gut microbiota of PCOS patients was characterized by a marked elevation of *Bacteroides vulgatus*, this bacterium could influence the metabolism of bile acids (e.g., glycodeoxycholic acid) and thus reduce the secretion of immune cytokines such as interleukin-22 (IL-22) that are beneficial to PCOS phenotype. The finding of this gut microbiota-bile acid-IL-22 axis also suggests that modulation of gut microbiota could be of great value for the treatment of PCOS (Qi et al., 2019).

As an important component of the gut microbiota, the viral community (referred to as the “virome”) is closely related to the health state of humans (Cadwell, 2015). In the gut, viral biological entities are thought to be in the same range as bacterial populations (Kim et al., 2011; Cadwell, 2015). They consist of a large number of prokaryotic viruses (mainly bacteriophages) and eukaryotic viruses (Shkoporov et al., 2019). The healthy human gut virome is highly specific to the individual and temporally stable (Reyes et al., 2010; Minot et al., 2013; Shkoporov et al., 2019). Gut viruses coevolved with gut bacteria to play an important role in intestinal homeostasis (Virgin, 2014; De Sordi et al., 2017). Recent studies have confirmed that enterovirus populations are associated with autoimmune diseases and inflammatory bowel diseases (Liang et al., 2021). Gut viruses may be the direct cause of diseases or indirectly affect the development of diseases by regulating the structure of the bacteria (Carding et al., 2017; Beller and Matthijnssens, 2019; Cao et al., 2022). In some diseases where the gut microbiota is seriously maladjusted, variation and maladjustment of the gut virome can also be observed (Santiago-Rodriguez and Hollister, 2019). The structure of the gut bacterial microbiota in PCOS patients is significantly different from that in healthy people (Liu et al., 2017; Torres et al., 2018; Qi et al., 2019), which may also lead to changes in the gut virome. Investigating these changes will help us understand how the gut virome relates to the gut microbiota. With the combination of metagenomic sequencing and virus sequence recognition algorithms, re-mining past metagenomic data may help deepen our understanding of the link between the gut virome and PCOS.

In this study, focusing on the alteration of the gut viral community in PCOS, we reanalyzed the deep fecal whole-metagenome dataset from a recent PCOS study comprising 50 patients and 43 healthy subjects (Qi et al., 2019). We profiled the whole gut virome from fecal metagenomes and identified the variations in gut viral composition and functions between patients and healthy individuals. We also performed correlation analyses between PCOS-associated gut viruses and bacteria. Our findings may contribute to a better understanding of PCOS and the development of novel therapeutic strategies.

## Materials and methods

### Data acquisition and processing

The metagenomic dataset of 93 samples from 50 PCOS patients and 43 healthy controls was downloaded from the Sequence Read Archive (NCBI-SRA) database under project accession no. PRJNA530971. Patients and controls were matched in their age, body mass index, and waist-hip ratio, as described in the original study (Qi et al., 2019). Quality control of raw metagenomic reads was performed using fastp (Chen et al., 2018). Briefly, low-quality (> 45 bases with quality score < 20, or > 5 “N” bases), low-complexity, and adapter-containing reads were removed, and the remaining reads were trimmed at the tails for low-quality (< Q20) or “N” bases. Human genomic reads were removed by mapping to the reference human genome (GRCh38) using Bowtie2 (Langmead and Salzberg, 2012).

### Gut virome profiling and analyses

To quantify the composition of the gut viral community in fecal metagenomic samples, we used a non-redundant gut virus catalog derived from over 10,000 publicly available fecal metagenomes from the Chinese population as a reference. Briefly, raw metagenomic reads were downloaded from public databases and assembled into contigs using MEGAHIT (Li et al., 2015) with the options “--kmer 21,33,55” (for samples with read length < 100 bp or less) or “--kmer 21,33,55,77” (for samples with read length > 100 bp). Potential viral sequences were identified from the contigs based on any of the following criteria: (1) contig whose viral genes were more than the number of microbial genes in CheckV (contigs with length < 10 kbp and estimated as low-quality or undetermined contigs were removed; Nayfach et al., 2021b); (2) contig with value of *p* < 0.01 and score > 0.90 in DeepVirFinder v1.0 (Ren et al., 2017); and (3) contig identified by VIBRANT v1.2.1 (Kieft et al., 2020) with default options. Viral sequences which shared 95% nucleotide identity across 75% of the sequence were then clustered into a viral operational taxonomic unit (vOTU) using the custom scripts. The resulting gut virus catalog contained the genome sequences of a total of 67,096 vOTUs with completeness > 50%, as estimated by CheckV (Nayfach et al., 2021b). Taxonomy annotation and prokaryotic host prediction of the vOTUs were performed according to the methods described in the previous studies (Yan et al., 2021; Li et al., 2022).

High-quality metagenomic reads of the samples of PCOS patients and healthy controls were mapped into the reference gut virus catalog using Bowtie2 with a nucleotide similarity threshold of 95% (Langmead and Salzberg, 2012). The abundance profile of vOTUs in each sample was generated by aggregating the number of reads mapped to each vOTU. The relative abundance of vOTUs was divided by the number of total mapped reads in each sample. The relative abundance profile at the viral family level was generated by aggregating the relative abundance of vOTUs assigned to the same family.

We calculated two diversity indexes to assess the richness and evenness of the vOTU composition for each sample. The number of observed vOTUs was defined as the count of unique vOTUs in each sample. Shannon’s diversity index was calculated using the *vegan* package (function *diversity*) in the R platform.

Viral proteins of vOTUs were predicted using Prodigal v2.6.3 (Hyatt et al., 2010). Functional annotation of viral proteins was performed based on the Kyoto Encyclopedia of Genes and Genomes (KEGG) database (Kanehisa et al., 2017) using DIAMOND with the options “--query-cover 50 --subject-cover 50-e 1e-5 --min-score 50 --max-target-seqs 50.” Each protein was assigned a KEGG orthology (KO) on the basis of the best-hit gene in the database. The viral auxiliary metabolic genes (AMGs) were identified according to the method described by a previous study (Kieft et al., 2020).

### Gut bacteriome profiling

The comprehensive Unified Human Gastrointestinal Genome (UHGG; Almeida et al., 2021) database of the human gut microbiome was used as a reference for bacteriome profiling of fecal metagenomes. The gut species of the UHGG database were taxonomically annotated using the Genome Taxonomy Database (GTDB; Chaumeil et al., 2019). High-quality metagenomic reads of the samples of PCOS patients and healthy controls were mapped into the UHGG database using Bowtie2 with a nucleotide similarity threshold of 95% (Langmead and Salzberg, 2012). To improve the comparison of samples with vastly different read counts, we randomly subsampled 20,000,000 mapped reads per sample to recalculate the read count of each gut species. The relative abundance of each gut species in every sample was its read count divided by 20,000,000. For genus-level profiles, the relative abundances of species with the same genus were added together to calculate the abundance for the corresponding genus.

### Statistical analyses

Statistical analyses were performed based on the R 4.0.1 platform. Principal coordinate analysis (PCoA) was performed with the R *vegan* package, based on the Bray–Curtis dissimilarity, and visualized *via* the R *ade4* package. Permutational multivariate analysis of variance (PERMANOVA, also known as *adonis* analysis) was performed with the R *vegan* package, and the *adonis p* value was generated based on 1,000 permutations. Random forest models were analyzed with the R *randomForest* package (1,000 trees). The performance of the predictive model was evaluated using receiver operator characteristic (ROC) analysis, which was performed with the R *pROC* package.

The Wilcoxon rank-sum test was used to measure significant differences between two different groups. The *q* value was used to evaluate the false discovery rate (FDR) for correction of multiple comparisons and was calculated based on the R *fdrtool* package (Strimmer, 2008), and q-values <0.05 were considered statistically significant.

Spearman’s correlation analysis was used to analyze the correlation between viruses and bacteria. Correlations with an absolute correlation coefficient *p* > 0.60 and Spearman’s correlation test *q* < 0.05 were shown in the correlation network. The network was visualized using Cytoscape (Su et al., 2014).

## Results

### Overview of the gut virome

**Figure 1:**
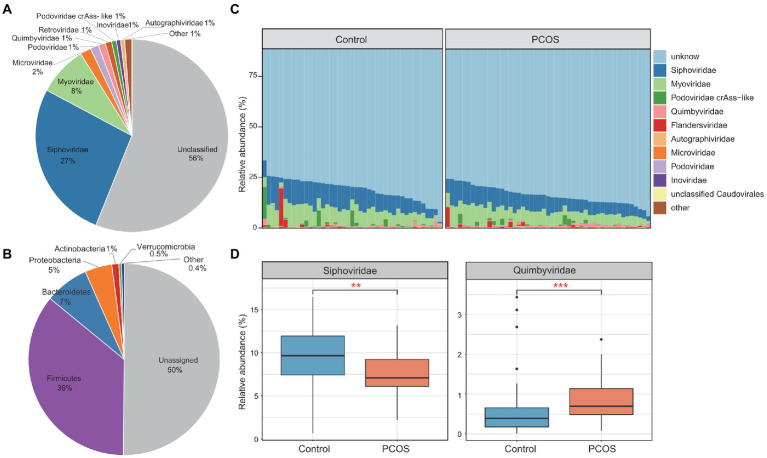
Summary of the gut virome in polycystic ovary syndrome (PCOS) patients and healthy controls. **(A)** The proportion of viral operational taxonomic units (vOTUs) that were assigned to viral taxa at the family level. **(B)** The proportion of vOTUs that are predicted to infect microbial hosts at the phylum level. **(C)** Bar plot showing the gut viral composition of all samples at the family level. Only the top 10 viral families with the highest abundance are shown. **(D)** Bar plot showing the relative abundance of differentially abundant viral families between the two groups. Boxes represent the interquartile range between the first and third quartiles, and the median (internal line). Whiskers denote the lowest and highest values within 1.5 times the range of the first and third quartiles, respectively; dots represent outlier samples beyond the whiskers. Wilcoxon rank-sum test with false discovery rate (FDR) correction: ^*^q < 0.05; ^**^q < 0.01; ^***^q < 0.001.

To investigate the gut viral characteristics in PCOS patients, we analyzed the metagenomic sequencing dataset from fecal samples of 50 patients and 43 healthy individuals (Qi et al., 2019). For all samples, the gut viral compositions were profiled by mapping the metagenomic reads to a non-redundant gut virus catalog constructed from publicly available fecal metagenomes of Chinese populations (see “Materials and methods”). A total of 21,204 vOTUs were observed and quantified in the dataset, which captured, on average, 10.1 ± 3.7 and 9.8% ± 4.0% of metagenomic reads from the samples of the patient and control groups, respectively. A total of 43.9% (9,308/21,204) of these vOTUs could be taxonomically assigned into 16 viral families. *Siphoviridae* and *Myoviridae* were dominant families, comprising 27 and 8% of all vOTUs, respectively (Figure 1A). The remaining vOTUs were members of *Microviridae*, *Podoviridae*, *Quimbyviridae*, *Retroviridae*, *Podoviridae_crAss-like*, *Inoviridae*, *Autographiviridae*, and a small number of *unclassified_Caudovirales*, *Gratiaviridae*, *Flandersviridae*, *Drexlerviridae*, *Metaviridae*, *Mitoviridae*, and *Herelleviridae* viruses. Additionally, we predicted the prokaryotic hosts of the vOTUs based on the comprehensive UHGG database (Almeida et al., 2021) and CRISPR interval sequences (see Methods). A total of 49.9% (10,572/21,204) of the vOTUs could be assigned to one or more microbial hosts (Figure 1B). Most of the predicted hosts were members of *Firmicutes* (dominated by *Ruminococcaceae* and *Lachnospiraceae* at the family level), *Bacteroidaceae* (*Bacteroidaceae* and *Prevotellaceae*), *Proteobacteria* (mainly *Enterobacteriaceae*), and *Actinobacteria* (mainly *Coriobacteriaceae*).

In the viral compositional profile, an average of only 20% of the total viral sequences could be captured by vOTUs belonging to known viral families (Figure 1C), suggesting that most viruses in the human gut are uncultured and need to be described. As expected, *Siphoviridae* (average relative abundance 9.5 ± 3.4%) and *Myoviridae* (7.6 ± 2.7%) were the most dominant families in all metagenomic samples, followed by *Podoviridae_crAss-like* (1.0 ± 2.4%), *Quimbyviridae* (0.8 ± 0.7%), and *Flandersviridae* (0.7 ± 2.5%). Compared with those of the healthy controls, the viral communities of the PCOS patients showed a significant increase in *Quimbyviridae* abundance and a significant decrease in *Siphoviridae* abundance (Wilcoxon rank-sum test, *q* < 0.05; Figure 1D).

### Characteristics of the gut virome in PCOS patients

**Figure 2:**
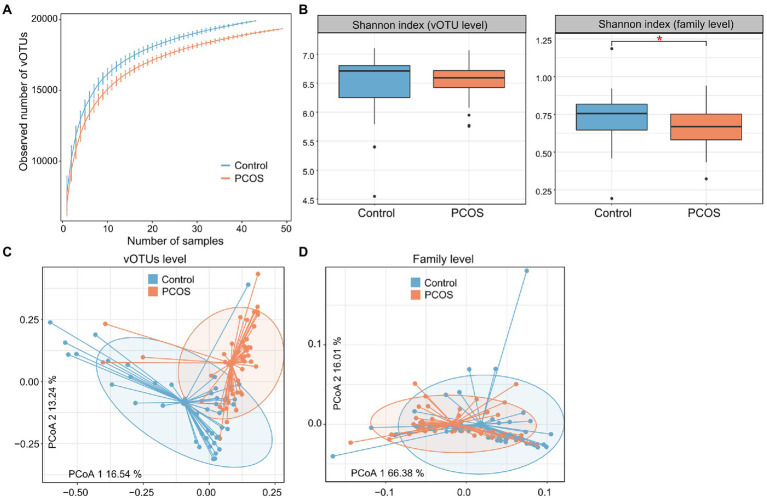
Diversity and PCoA analyses of the gut virome. **(A)** Rarefaction analysis showed an increase in the number of vOTUs observed as the number of random samples increased. **(B)** Boxplot showing the Shannon index of the gut virome of all samples at the vOTU and family levels. Boxes represent the interquartile range between the first and third quartiles and the median (internal line). Whiskers denote the lowest and highest values within 1.5 times the range of the first and third quartiles, respectively; dots represent outlier samples beyond the whiskers. **(C,D)** PCoA analysis of the Bray–Curtis distance of the gut virome at the vOTU **(C)** and family **(D)** levels. Samples are shown at the first and second principal coordinates (PC1 and PC2), and the ratio of variance contributed by these two PCs is shown. Ellipsoids represent a 95% confidence interval surrounding each group.

Rarefaction curve analysis showed that (1) the curve was approximately saturated under 20 samples in each group, and (2) the vOTU richness was significantly lower in the PCOS patients than in healthy controls at the same sample sizes (*p* = 0.008; Figure 2A). We then assessed the within-sample diversity of the gut viromes using Shannon’s diversity index. PCOS patients showed a slightly but not significantly lower Shannon index than healthy subjects at the vOTU level, whereas, at the viral family level, this index was significantly lower in PCOS patients than in controls (Figure 2B; Supplementary Table S1).

Next, we carried out PCoA based on the Bray–Curtis distance to further investigate the differences in gut viral communities between PCOS patients and healthy controls and revealed a clear separation between them at both the vOTU and family levels (Figures 2C,D). PERMANOVA also supported that the gut virome was significantly different between PCOS patients and healthy controls, with effect sizes of 7.7% (*adonis p* < 0.001) and 5.9% (*adonis p* < 0.001) at the vOTU and family levels, respectively.

### Identification of PCOS-associated gut viral signatures

**Figure 3:**
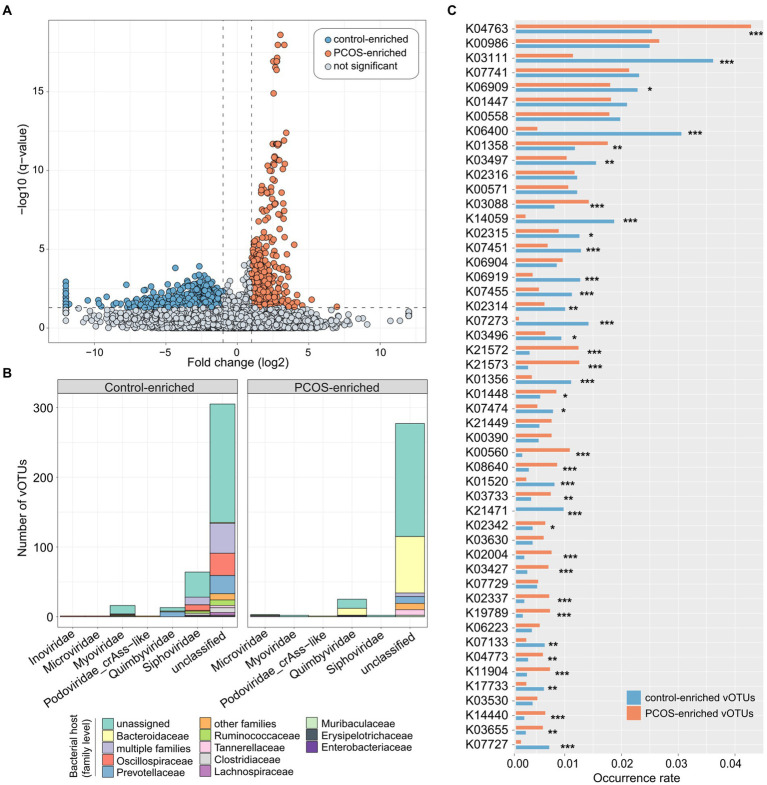
Polycystic ovary syndrome (PCOS)-associated viral signatures. **(A)** Volcano map showing the fold change and *q*-values of all vOTUs. vOTUs whose absolute value of fold change greater than 2 and *q*-value less than 0.05 were considered to be significantly different between PCOS patients and healthy controls, represented by blue and orange dots in the figure, respectively. **(B)** Bar accumulation plots showing the taxonomical and predicted host distributions of vOTUs enriched in the PCOS and control groups. Viruses that predicted to infect multiple bacterial families are labeled as “multiple families.” **(C)** Bar plot showing the occurrence rates of KEGG orthologs (KOs) in the PCOS-associated vOTUs. Only the top 50 KOs with the highest occurrence rates are shown. The significance level was calculated by Fisher’s exact test: ^*^q < 0.05, ^**^q < 0.01, and ^***^q < 0.001.

To explore the gut viral signatures of PCOS, we compared the viral composition between PCOS patients and healthy controls at the vOTU level. A total of 1,089 vOTUs were identified with significant differences in relative abundance between the two groups (Wilcoxon rank-sum test with FDR-correction *q* < 0.05,|fold-change| > 2; Supplementary Table S2). Among these, 455 vOTUs were enriched in the virome of PCOS patients, and 634 vOTUs were enriched in controls (Figure 3A). Most (81.5%) of these PCOS-associated vOTUs could not be classified into known viral families, while the remaining taxonomically annotated vOTUs were members of *Siphoviridae* (containing 113 vOTUs), *Quimbyviridae* (50 vOTUs), *Myoviridae* (23 vOTUs), *Microviridae* (8 vOTUs), *Podoviridae_crAss-like* (5 vOTUs), *Gratiaviridae* (1 vOTU), and *Inoviridae* (1 vOTU; Figure 3B). Thirty-four of 50 PCOS-associated *Quimbyviridae* vOTUs were enriched in the gut virome of PCOS patients compared with those in the gut virome of controls, while only 16 *Quimbyviridae* vOTUs were depleted in patients. Conversely, 108 of 113 PCOS-associated *Siphoviridae* vOTUs and 21 of 23 PCOS-associated *Microviridae* vOTUs were enriched in the gut virome of healthy controls. Regarding the host, most of the differentially abundant gut vOTUs were bacteriophages that were predicted to infect bacterial hosts such as *Bacteroidaceae*, *Oscillospiraceae*, *Prevotellaceae*, and *Ruminococcaceae* (Figure 3B; Supplementary Table S2). The PCOS-enriched vOTUs were concentrated in *Bacteroidaceae* phages (35.2% of 455 PCOS-enriched vOTUs), while the control-enriched vOTUs were more likely to be distributed in *Oscillospiraceae* (10.7% of 634 control-enriched vOTUs) and *Prevotellaceae* (9.5%) phages.

To elucidate the functional potential of the PCOS-associated viruses, we performed functional annotation of the vOTUs based on the KEGG database (Kanehisa et al., 2017). A total of 87,357 protein-coding genes were predicted from 1,089 PCOS-associated vOTUs, and 16.8% (14,691/87,357) of these genes matched the corresponding KEGG orthologs. We then focused on the top 50 KOs (which represented 41.6% of all annotated genes) with the highest occurrence rates in all PCOS-associated vOTUs. Thirty-seven of 50 KOs had significantly different occurrence rates between PCOA and healthy control groups, of which 18 were more frequent in PCOS-enriched vOTUs and 19 in control-enriched vOTUs (Figure 3C). The PCOS-enriched vOTUs encoded functions, such as integrase/recombinase (K04763), ATP-dependent protease (K01358), RNA polymerase sigma-70 factor (K03088), starch-binding outer membrane proteins (K21572/K21573), and thymidylate synthase (K00560), while the control-enriched vOTUs encoded more functions involving single-stranded DNA-binding protein (K03111), site-specific DNA recombinase (K06400), chromosome-partitioning protein (K03497), integrase (K14059), 5-methylcytosine-specific restriction enzyme (K07451), and DNA primase/helicase (K06919; Supplementary Table S3).

### Classification of PCOS state using gut viral signatures

**Figure 4:**
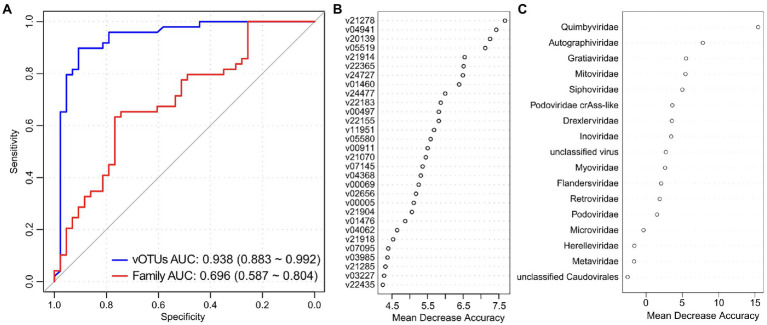
Gut virome-based classification of the PCOS patients and controls. **(A)** Random forest models for discriminating PCOS patients and healthy controls based on gut viral profiles at the vOTU and family levels. The AUC and 95% CI are shown. **(B,C)** Mean decrease in accuracy showing the contribution of the top 30 vOTUs **(B)** and all viral families **(C)** in the random forest models.

We used the random forest regression model with 5-fold cross-validation to assess the performance of gut viral signatures in recognizing PCOS status. A model trained based on the relative abundances of the PCOS-associated vOTUs obtained high identification ability in classifying PCOS patients and healthy controls, with an AUC of 0.938 (95% CI, 0.993–0.992, Figure 4A). Several PCOS-enriched vOTUs, such as v21278, v21914, v05519, v20139, and v04941, featured the highest score for the discrimination of patients and controls (Figure 4B), and the former 4 vOTUs were predicted to be Bacteroidaceae phages. On the other hand, a model trained by all viral families resulted in a significant decline in discrimination power, with an AUC of 0.696 (95% CI, 0.587–0.804). The PCOS-enriched family *Quimbyviridae* had the highest contribution in this model (Figure 4C). Taken together, these findings suggested that gut viral signatures have the potential for the differentiation of PCOS patients from controls.

### Correlations between gut viral signatures and bacteria

**Figure 5:**
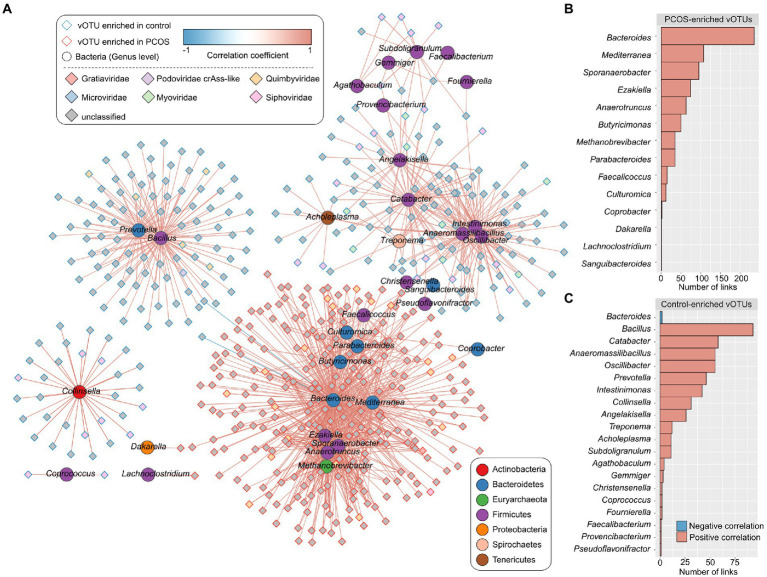
Correlation analysis between PCOS-associated vOTUs and gut bacteria (genus level). **(A)** Network showing the correlations of the vOTUs and bacterial genera. The Spearman’s correlation coefficient was used to evaluate the correlation, and correlations with an absolute correlation coefficient  > 0.60 and Spearman’s correlation test *q* < 0.05 are shown in the network. Blue and red lines represent negative and positive correlations, respectively. **(B,C)** Bar charts showing the number of links between bacterial genera and PCOS-enriched vOTUs **(B)** and between bacterial genera and control-enriched vOTUs **(C)**.

To explore the relationship between viral signatures and bacteria in the gut ecosystem, we mapped the metagenomic sequencing dataset of PCOS patients and healthy controls to the gut prokaryotic UHGG database (see “Materials and methods”) and obtained a genus-level bacterial profile of all samples (representing 85.8% of metagenomic reads for all samples). Spearman’s correlation coefficient analysis was used to evaluate the interactions between 1,089 PCOS-associated vOTUs and 287 bacterial genera. This analysis revealed a large virus–bacterium interaction network (Figure 5A) consisting of a total of 1,184 co-occurrence correlations between 514 vOTUs and 33 bacterial genera (Supplementary Table S4). Several bacterial taxa, such as *Bacteroides*, *Mediterranea*, and *Sporanaerobacter*, were positively correlated with the highest number of PCOS-enriched vOTUs in the network, while other bacteria, such as *Bacillus*, *Catabacter*, *Anaeromassilibacillus*, *Oscillibacter*, *Prevotella*, and *Intestinimonas*, were positively correlated with the highest number of healthy control-enriched vOTUs (Figures 5B,C); these findings suggest the potential central roles of these bacterial genera in terms of interacting with PCOS-associated viruses.

## Discussion

Polycystic ovary syndrome (PCOS) has been described as a serious health problem for women of childbearing age. There is abundant evidence that the gut microbiota contributes significantly to the development of PCOS (Liu et al., 2017; Torres et al., 2018; Qi et al., 2019). Deep metagenomic sequencing provides an opportunity to explore the links between the alteration of the gut viral community and disease (Gregory et al., 2020; Camarillo-Guerrero et al., 2021; Nayfach et al., 2021b). In this study, using a metagenomic-based methodology, we characterized the diversity, composition, and functional repertoire of the gut viromes of 50 PCOS patients and 43 healthy women. Multivariate analyses revealed that the overall structure of the gut virome was changed in PCOS patients, with considerably high effect size, consistent with previous observations for the PCOS bacteriome (Guo et al., 2016; Liu et al., 2017; Qi et al., 2019; Belkova et al., 2020). Our study strengthened the previous bacteriome study on PCOS (Qi et al., 2019) by adding new information about the virome. Furthermore, we observed significant differences in viral community dysbiosis, taxonomic shifts, and functional changes between PCOS and control viromes.

The within-sample diversity index of the gut virome of PCOS patients was significantly lower than that of healthy women, which may be related to the decrease in the diversity and total amount of bacteria in these patients (Qi et al., 2019). Reduction of gut viral diversity and alteration of virome structure had also been observed in patients with other disorders, such as inflammatory bowel disease (Clooney et al., 2019; Zuo et al., 2019), colorectal cancer (Nakatsu et al., 2018), or liver diseases (Lang et al., 2020). Thus, our findings highlighted a remarkable dysbiosis of the gut virome in PCOS patients.

*Siphoviridae*, the most dominant viral family of the normal human gut viral community (Zuo et al., 2020; Tomofuji et al., 2021; Lee et al., 2022), was found to be less distributed in the gut virome of PCOS patients than in that of controls. *Siphoviridae* is a type of double-stranded DNA (dsDNA) virus that is known to be rich in temperate phages and infects a wide range of gut bacteria, such as *Bacillus*, *Enterococcus*, *Clostridium*, *Streptococcus*, *Lactobacillus*, and *Escherichia* (Sekulovic et al., 2014; Adriaenssens et al., 2015; Chehoud et al., 2016). At the vOTU level, 108 *Siphoviridae* vOTUs were identified as enriched in the gut virome of healthy controls and were predicted to infect *Oscillospiraceae*, *Prevotellaceae*, and *Ruminococcaceae*, whereas only five *Siphoviridae* vOTUs were enriched in PCOS patients. On the other hand, the most representative PCOS-enriched viral taxon was *Quimbyviridae*, which accounted for over 2-fold of the relative abundance in the gut virome of PCOS patients and contained 34 vOTUs that were significantly enriched in these patients. *Quimbyviridae* is a recently described viral family with a highly abundant and wide prevalence in the human gut and is suspected to be an obligate lytic phage (Benler et al., 2021). The known bacterial hosts of PCOS-enriched *Quimbyviridae* members were mostly Bacteroidaceae. Collectively, these findings suggested that a low level of *Siphoviridae* viruses and overrepresentation of Bacteroidaceae-infected *Quimbyviridae* viruses in the gut environment may be considered risk factors for PCOS.

Functional comparison between PCOS-enriched and control-enriched vOTUs identified a large number of viral functions that are potentially associated with the disease. In accordance with previous observations (Nayfach et al., 2020), most of these functions were typical viral functions, such as integrase, recombinase, and RNA polymerase. Additionally, some functions involving viral auxiliary metabolism (e.g., thymidylate synthase K00560) were also identified with a significantly different frequency between the PCOS- and control-enriched vOTUs. Based on this information, however, the potential correlation between viral function and PCOS still needs to be explored.

Our study further provided gut viral biomarkers for PCOS discrimination and achieved an AUC of 0.938 for identifying disease status based on the differentially abundant vOTUs. This discriminatory power was higher than that of the prediction models based on genotypic or phenotypic markers (Cui et al., 2013; McAllister et al., 2015; Kodipalli and Devi, 2021). Thus, the gut virome showed good potential for the prediction and early diagnosis of PCOS; however, systematic investigations of key viral or functional markers identified here might be helpful in the future.

There were limitations in this study. One of these was the lack of information on the individuals’ phenotypic factors, such as sex, age, body mass index, diet, and lifestyle. The relationship between host factors and the gut virome is still unclear (Shkoporov et al., 2019); thus, a larger well-described cohort of PCOS patients is needed to further understand the relationship between the gut virome and PCOS. On the other hand, as PCOS is a highly complex and heterogeneous disease, it is currently infeasible to draw any conclusions about the causal relationships between the gut virome and PCOS, and direct experimental studies (e.g., animal model studies) are needed to show the causality of proposed viral or functional signatures.

## Conclusion

To our knowledge, this is the first study investigating the gut virome in patients with PCOS. Our findings revealed additional important information on the correlations between the gut viral community and PCOS and extended previous knowledge. Our results suggested that the gut virome is dysregulated in patients with PCOS and may play a potential role in the pathogenesis of the disease. The viral taxonomic signatures, virus–bacteria associations, and functional signatures identified in this study provided potential markers for PCOS prediction and intervention.

## Data availability statement

The original contributions presented in the study are included in the article/Supplementary material, further inquiries can be directed to the corresponding author.

## Ethics statement

Ethical review and approval was not required for the study on human participants in accordance with the local legislation and institutional requirements. Written informed consent for participation was not required for this study in accordance with the national legislation and the institutional requirements.

## Author contributions

LH and WS proposed the idea, designed the study, and wrote and improved the manuscript. LH, XW, SG, YL, and WS performed the study. LH, PZ, GH, ZD, and WS processed the experimental data and performed the analysis and interpretation of the results. All authors contributed to the article and approved the submitted version.

## Funding

This work was supported by the Science and Technology Innovative Project of Bao’an, Shenzhen (2019JD318) and COVID-19 Research & Application Project, Shenzhen Bao’an Chinese Medicine Developing Fund (2020KJCX-KTYJ-76).

## Conflict of interest

The authors declare that the research was conducted in the absence of any commercial or financial relationships that could be construed as a potential conflict of interest.

## Publisher’s note

All claims expressed in this article are solely those of the authors and do not necessarily represent those of their affiliated organizations, or those of the publisher, the editors and the reviewers. Any product that may be evaluated in this article, or claim that may be made by its manufacturer, is not guaranteed or endorsed by the publisher.
